# Glycosylphosphatidylinositol Anchor Biosynthesis Pathway-Related Protein GPI7 Is Required for the Vegetative Growth and Pathogenicity of *Colletotrichum graminicola*

**DOI:** 10.3390/ijms23062985

**Published:** 2022-03-10

**Authors:** Jie Mei, Na Ning, Hanxiang Wu, Xiaolin Chen, Zhiqiang Li, Wende Liu

**Affiliations:** 1 State Key Laboratory for Biology of Plant Diseases and Insect Pests, Institute of Plant Protection, Chinese Academy of Agricultural Sciences, Beijing 100193, China; mj1992yx@126.com (J.M.); ningna960328@163.com (N.N.); wuhanxiang@caas.cn (H.W.); 2 Key Laboratory of Agricultural Microbiology, College of Agriculture, Guizhou University, Guiyang 550025, China; 3 The Provincial Key Lab of Plant Pathology of Hubei Province, College of Plant Science and Technology, Huazhong Agricultural University, Wuhan 430070, China; chenxiaolin@mail.hzau.edu.cn

**Keywords:** *C. graminicola*, GPI anchoring, cell wall integrity, vegetative growth, pathogenicity

## Abstract

Glycosylphosphatidylinositol (GPI) anchoring is a common post-translational modification in eukaryotic cells and has been demonstrated to have a wide range of biological functions, such as signal transduction, cellular adhesion, protein transport, immune response, and maintaining cell wall integrity. More than 25 proteins have been proven to participate in the GPI anchor synthesis pathway which occurs in the cytoplasmic and the luminal face of the ER membrane. However, the essential proteins of the GPI anchor synthesis pathway are still less characterized in maize pathogen *Colletotrichum graminicola*. In the present study, we analyzed the biological function of the GPI anchor synthesis pathway-related gene, Cg*GPI7,* that encodes an ethanolamine phosphate transferase, which is localized in ER. The vegetative growth and conidia development of the *ΔCgGPI7* mutant was significantly impaired in *C. graminicola.* and qRT-PCR results showed that the transcriptional level of *CgGPI7* was specifically induced in the initial infection stage and that the pathogenicity of *ΔCgGPI7* mutant was also significantly decreased compared with the wild type. Furthermore, the *ΔCgGPI7* mutant displayed more sensitivity to cell wall stresses, suggesting that *CgGPI7* may play a role in the cell wall integrity of *C. graminicola*. Cell wall synthesis-associated genes were also quantified in the *ΔCgGPI7* mutant, and the results showed that chitin and β-1,3-glucans synthesis genes were significantly up-regulated in *ΔCgGPI7* mutants. Our results suggested that *CgGPI7* is required for vegetative growth and pathogenicity and might depend on the cell wall integrity of *C. graminicola*.

## 1. Introduction

Glycosylphosphatidylinositol (GPI) anchoring is a common post-translational modification in the endoplasmic reticulum (ER) luminal of eukaryotic cells and is responsible for anchoring proteins to the outside of the plasma membrane [1,2,3]. GPI-anchored proteins (GPI-APs) exist ubiquitously in fungi, plants, and animals, and represent 0.5% of total proteins in most eukaryotic species and even 1% of plant proteins [4]. To date, GPI-anchored proteins have been found to play critical roles in signaling, cell growth, immune response, cell development, and other life processes [5]. GPI anchor synthesis is indispensable for nematode germline development in *Caenorhabditis elegans* [6]. In mammalians such as humans, defects in GPI anchor synthesis have triggered a rare disease called paroxysmal nocturnal hemoglobinuria which affects the normal protection of complement factors by erythrocytes [7]. Moreover, GPI-APs, such as the prion protein PrP^C^, the folate receptor alpha, and the urokinase plasminogen activator receptor, have been proven to be involved in human neurodegenerative diseases and cancer [8,9,10]. In fungi, GPI-APs have been widely studied and reported to serve a variety of functions, from basic cell wall biosynthesis and remodeling to host recognition and immune evasion [3,11,12]. On the basis of its biological importance, the GPI anchor has been recently confirmed to be a promising antifungal target [13].

In eucaryotes, GPI anchoring happens in the ER luminal and the whole process involves GPI anchor biosynthesis and covalent linkages to the nascent peptides. In *Saccharomyces cerevisiae*, the initial step of GPI anchor synthesis is the addition of N-acetyl-glucosamine (GlcNAc) to phosphatidylinositol (PI) catalyzed by the GPI-N-acetylglucosaminyltransferase (GPI-GnT) complex. This transfer reaction occurs at the cytosolic side of the ER and is mediated by a multi-subunit enzyme complex which is composed of six phosphatidylinositol GPI-GnTs (GPI1, GPI2, GPI3, GPI15, ERI1, and GPI19) [14,15,16,17]. The acetyl group of GlcNAc-PI is subsequently removed by N-acetylglucosaminyl-phosphatidylinositol de-N-acetylase GPI12, and the resulting GlcNH_2_-PI is translocated to the luminal side of ER by transfer protein Gwt1 [18,19]. In the ER lumen, four mannoses and three phosphorylethanolamines (EtN-P) are stepwise added to the intermediate GlcNH_2_-PI and form the core structure of a GPI anchor (Figure S1). The corresponding reactions are catalyzed by the four mannosyltransferases (GPI14, GPI18, GPI10, and SMP3) and the three ethanolamine phosphate transferases (MCD4, GPI13, and GPI7) [20,21,22,23,24,25,26]. The addition of the GPI-lipids to new GPI proteins in ER is also mediated by a multi-enzyme complex composed of five transamidases (GAA1, GPI8, GPI16, GPI17, and GAB1) [3]. In *S. cerevisiae* and mammalians, all the five proteins have been proven to be essential for the nucleophilic attack on the ω site residue at the carboxyl terminal of GPI-anchored proteins. The recognition and cleavage of the ω site by transamidase complex causes the exposure of attachment sites that are covalently linked by mature GPI anchors [27,28]. So far, GPI anchoring involves many essential biological functions, such as embryogenesis, fertilization, neurogenesis, and immune response, in mammalians, as proven by the mutation of GPI lipid remodeling-related genes [29]. As with mammalians, GPI anchoring is also essential for viability in *S. cerevisiae*, since any deletion of the key proteins in GPI anchor biogenesis is lethal [30,31].

In recent years, functional studies of GPI anchor biosynthesis-related genes have revealed that GPI anchoring is essential for the development and virulence of fungal pathogens. In *Aspergillus fumigatus*, deletion of *GPI3* caused the defect of morphogenesis and virulence and deletion of *GPI7* affected the transportation of cell wall GPI anchored proteins and polarized growth [32,33]. In *Magnaporthe oryzae*, *GPI7* was proved essential for appressorial penetration and immune evasion during infection. This study proved that deletion of *GPI7* caused not only the defective cell wall integrity but also the exposure of chitin and β-1,3-glucan to the host immune system which well revealed the mechanism of GPI anchoring mediated pathogen-plant interaction [12].

*Colletotrichum graminicola* is the causal agent of maize anthracnose leaf blight and stalk rot, which leads to annual losses of up to USD 1 billion in the USA [34,35]. Based on the economic importance and the typical hemibiotrophic characteristics, *C. graminicola* has become an ideal model fungus for studying maize–pathogen interactions. However, the biological properties and relative pathogenic mechanisms of this maize pathogen remain poorly understood. In this study, we performed a systematic comparison of the GPI anchor biosynthesis pathway in *C. graminicola* and *S. cerevisiae* using a homologous blast method. The result showed all the 23 GPI anchor pathway proteins in *S. cerevisiae* have a unique homolog in *C. graminicola*, except *GPI15* (Figure S1). Three ethanolamine phosphate transferases (MCD4, GPI13, and GPI7) that are responsible for the addition of EtH-Ps to the GPI anchor intermediate in *C. graminicola* were selected for a functional exploration in this study. In *S. cerevisiae*, the deletion of *MCD4* and *GPI13* is lethal [22,23]; correspondingly, we did not successfully obtain the *MCD4* and *GPI13* gene knockout mutants in *C. graminicola*, except for *GPI7.* The subsequent study indicates that Cg*GPI7* is required for fungal vegetative growth, conidia development, cell wall integrity, and virulence.

## 2. Results

### 2.1. Identification of GPI Anchor Synthesis Pathway Proteins in C. graminicola

A previous study identified 23 GPI anchor biosynthesis proteins in *S. cerevisiae* [3]. To verify the conservation of this process, we used the *S. cerevisiae* GPI anchor biosynthesis pathway proteins as the BLAST queries to search the maize pathogen *C. graminicola* protein database. All the putative GPI anchor biosynthesis pathway orthologous proteins but GPI15 of *S. cerevisiae* were present in *C. graminicola* (Figure S1, Table S2). This result demonstrated that the GPI anchor synthesis pathway in *C. graminicola* is highly homologous to that of *S. cerevisiae*, except for the initial step.

### 2.2. Phylogenetic Analysis of CgEPTs in C. graminicola

**Figure 1:**
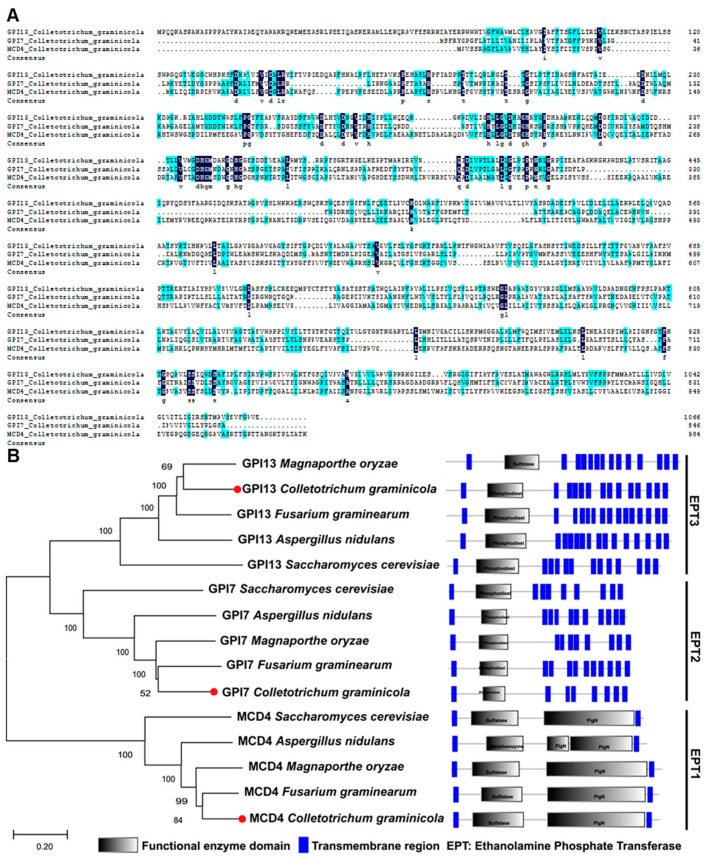
Phylogenetic analysis of GPI ethanolamine phosphate transferase family proteins. (**A**) Multiple alignments of ethanolamine phosphate transferases CgGPI13, CgGPI7, and CgMCD4 in *C. graminicola*. Total sequence identity was 21.06%. (**B**) Phylogenetic tree of GPI ethanolamine phosphate transferases from different fungal species. EPT, ethanolamine phosphate transferase.

A previous study showed the knockdown of GPI anchor synthesis pathway genes *GPI8*, *GPI12*, and *GAA1* seriously affected the cell wall integrity and pathogenicity of *C. graminicola* [36], which demonstrated that GPI anchoring is important in *C. graminicola*. In this study, we focused on the functional exploration of three GPI ethanolamine phosphate transferase (EPT) genes, *MCD4*, *GPI7*, and *GPI13*, in *C. graminicola*. The *CgMCD4*, *CgGPI7*, and *CgGPI13* encode three putative proteins, EPT1, EPT2, and EPT3, in *C. graminicola*, with the amino acids sequence lengths of 985 aa, 846 aa, and 1066 aa, respectively. Multi-sequence alignment showed that the three EPT family members share only 21.6% of their identity (Figure 1A). Then, we identified the homologous proteins of CgMCD4, CgGPI7, and CgGPI13 in other filamentous pathogenic fungi. Multiple sequence alignment and phylogenetic tree analyses confirmed that homologous proteins of MCD4, GPI7, and GPI13 were highly conserved in these species and exhibit different subfamily clusters (Figure 1B and Figure S2–S4).

### 2.3. CgGPI7 Is Essential for Vegetative Growth and Conidia Development of C. graminicola

**Figure 2:**
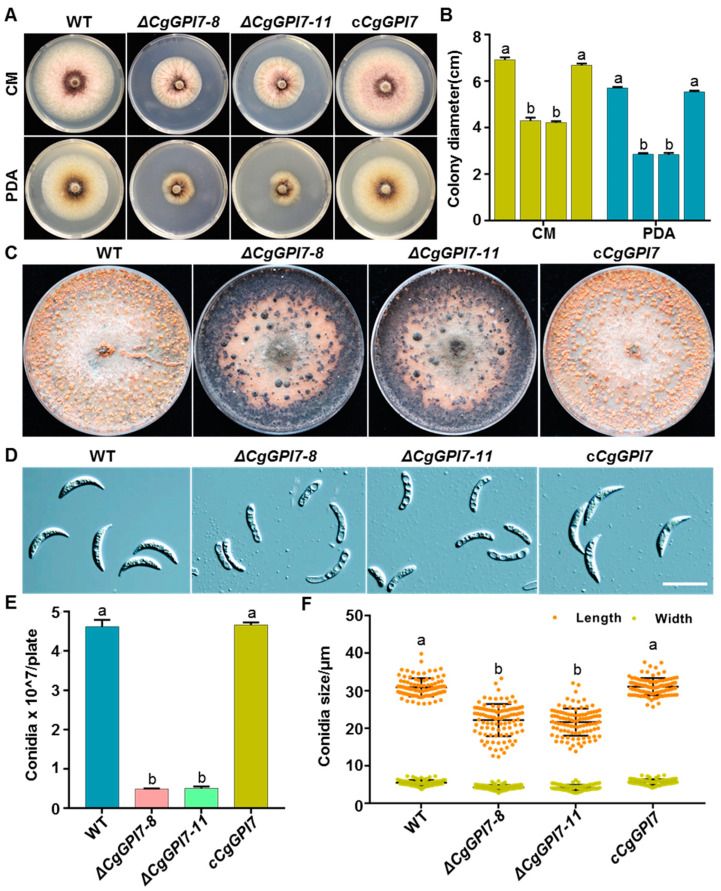
Biological phenotypic analysis of *CgGPI7* knockout mutants. (**A**) Colony morphology of wild type, *ΔCgGPI7-8*, *ΔCgGPI7-11,* and c*CgGPI7* grown on CM and PDA medium. (**B**) Statistical analysis of the colony diameters. Bar indicates the standard deviation of three replicates and the small letters indicate a significant difference (*p* < 0.01). (**C**) Sporulation of indicated strains on OA medium. (**D**) Conidia morphology of indicated strains. Scale bars = 25 μm. (**E**) Statistical analysis of the sporulation of indicated strains. Bar indicates the standard deviation of three replicates and the small letters indicate a significant difference (*p* < 0.01). (**F**) Statistical analysis of the conidia length and width of indicated strains. Bar indicates the standard deviation of 100 replicates and the small letters indicate a significant difference (*p* < 0.01).

To identify the functions of the three *EPT* genes, we firstly performed the gene knockout using the split PCR mediated gene disruption method in *C. graminicola* (Figure S5A). Unfortunately, we have not successfully obtained the *CgMCD4* and *CgGPI13* gene knockout mutants after four independent gene disruption experiments. This might be consistent with the previous report that the deletion of *MCD4* and *GPI13* is lethal in *S. cerevisiae* [22,23]. Fortunately, three independent *GPI7* knockout mutants were obtained, and *ΔCgGPI7-8* and *ΔCgGPI7-11* were used for further study. Additionally, *ΔCgGPI7-11* was used to generate the gene complementation strains. All the gene deletion and complementary strains were identified via PCR and the quantification of the transcript level (Figure S5B,C). To analyze the function of *CgGPI7*, we firstly compared the radial growth of *ΔCgGPI7-8* and *ΔCgGPI7-11* with the wild-type and complementary strain c*CgGPI7* on CM and PDA plates, respectively. Results showed that the growth rate of *ΔCgGPI7* mutant strains was significantly decreased compared with the wild-type and *cCgGPI7* strains (Figure 2A,B). Interestingly, the *ΔCgGPI7* mutants exhibit different colony and conidial morphology compared with the wild-type and *cCgGPI7* strains when cultured on OA plates (Figure 2C,D). Normally, the mature colony and conidia are visible and distributed in piles on the surface of the OA plate, just like the wild type and *cCgGPI7* in Figure 2C. However, for *ΔCgGPI7* mutants, the edge of the colony showed black mycelium accumulation, and no visible conidia piles were exhibited on the OA plates. Correspondingly, the conidiation of *ΔCgGPI7* mutants was significantly reduced and the conidia length was shorter than that of the wild-type and *cCgGPI7* strains (Figure 2E,F). These results demonstrated that *CgGPI7* is essential for the vegetative growth and conidia development of *C. graminicola*. 

### 2.4. Gene Expression Pattern of CgGPI7 in C. graminicola

**Figure 3:**
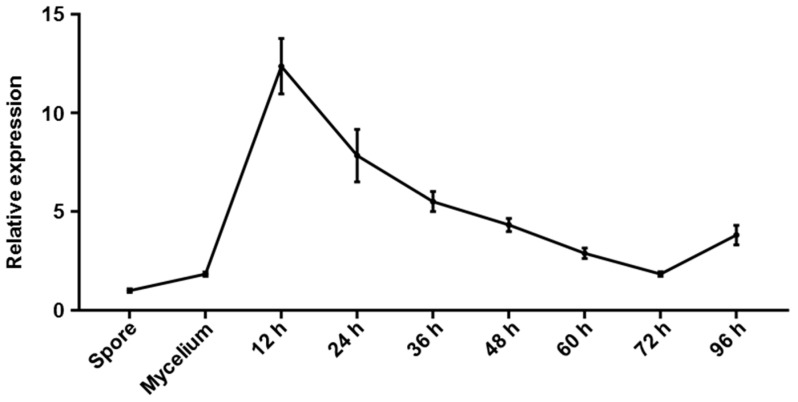
Expression pattern of *CgGPI7* at different stages. Samples from vegetative (spore and mycelium) and invasive stages (12, 24, 36, 48, 72, and 96 hpi) of infected maize leaves were collected and the expression patterns of *CgGPI7* were quantified via qRT-PCR. *CgGPI7* was especially induced at the initial infection stages. The bar represents the standard deviation of the three technical duplications.

To further explore the function of *CgGPI7*, we performed the qRT-PCR method to detect the transcriptional levels of *CgGPI7* at different development stages, including conidia and hyphae of *C. graminicola* in vegetative development stages. A conidial suspension of *C. graminicola* was sprayed on the maize seedlings and the inoculated leaf tissues were collected at 12 h, 24 h, 36 h, 48 h, 60 h, 72 h, and 96 h post-inoculation in the invasive growth stage. As shown in Figure 3, the expression level of the *CgGPI7* gene was significantly up-regulated at the initial infection stage (12 hpi) and kept a relatively high expression level at the subsequent infection stages compared with the conidia stage. These results indicated that *CgGPI7* might play a role in the pathogenicity of *C. graminicola*.

### 2.5. CgGPI7 Localized in the Endoplasmic Reticulum (ER)

**Figure 4:**
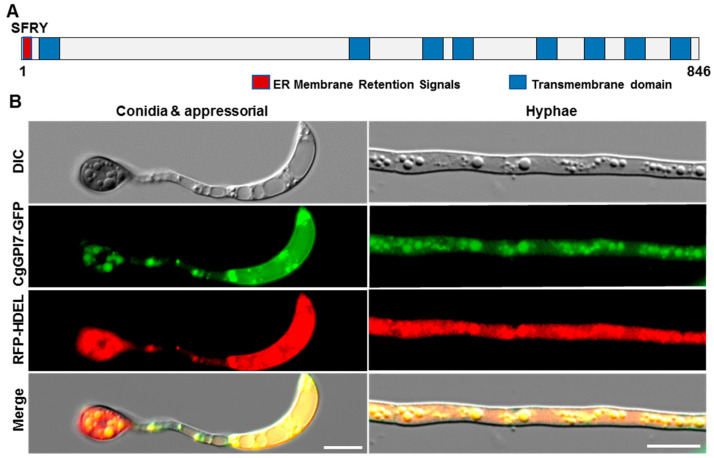
CgGPI7 located in the endoplasmic reticulum in *C. graminicola*. (**A**) Subcellular localization prediction showed CgGPI7 protein harboring an ER membrane retention signal at the N-terminal of the amino acid sequence. (**B**) CgGPI7-GFP and the ER marker RFP-HDEL were co-located at the ER in the conidia, the appressorium, and the vegetative hyphae of *C. graminicola*. Scale bars = 10 μm.

GPI anchoring usually happened in the endoplasmic reticulum (ER) and the GPI anchor synthesis pathway-related proteins were predominately localized at the ER membrane. To identify whether CgGPI7 was located in the ER membrane of *C. graminicola*, we firstly performed a subcellular localization prediction of CgGPI7 using online software PSORT (https://www.genscript.com/psort.html, accessed on 1 February 2021). The result showed that CgGPI7 harbored an ER membrane retention signal SFRY at the N-terminal of the amino acid sequence (Figure 4A). To further verify this result, we co-transformed the CgGPI7-GFP and ER maker RFP-HDEL to the protoplast of *ΔCgGPI7* and obtained the corresponding transformants with the complete CgGPI7-GFP and RFP-HDEL sequence. The *ΔCgGPI7/CgGPI7-GFP/RFP-HDEL* recovered the phenotype of *ΔCgGPI7* to the wild-type strain. Then, we observed the geminated conidia and vegetative hyphae of *ΔCgGPI7/CgGPI7-GFP/RFP-HDEL* under a Leica DM6B fluorescence microscope, and the results showed that CgGPI7-GFP and RFP-HDEL were co-located in the ER (Figure 4B). This result suggested that CgGPI7 was localized in the ER in *C. graminicola*.

### 2.6. CgGPI7 Is Essential for Pathogenicity of C. graminicola

**Figure 5:**
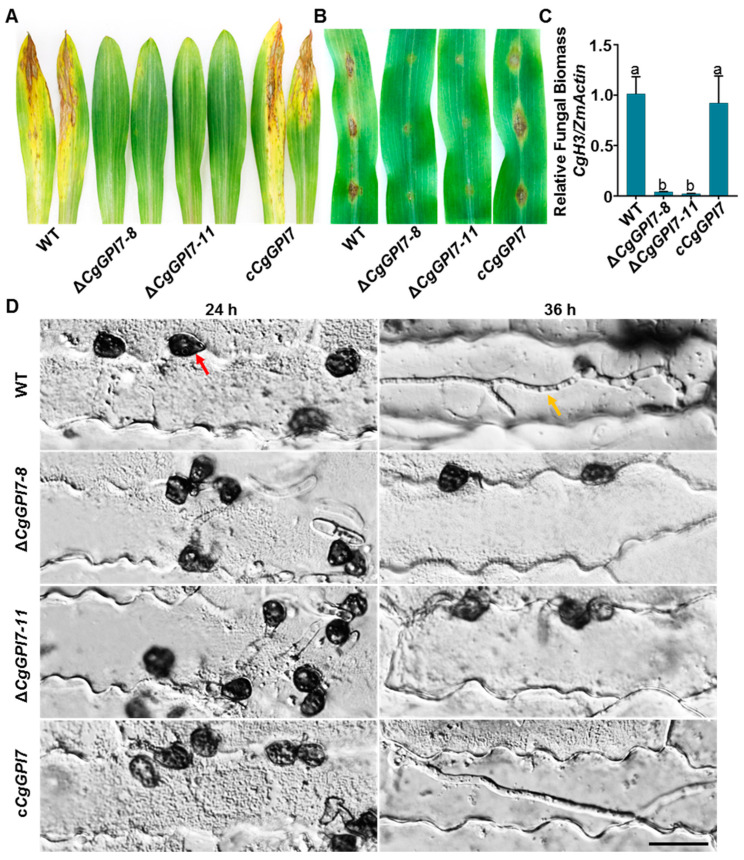
*ΔCgGPI7* mutants exhibit reduced pathogenicity. (**A**) Pathogenicity test by spray inoculation on live maize seedings. Pictures of inoculated leaves were taken at 5 dpi. (**B**) Pathogenicity test on detached maize seedling leaves. Pictures of inoculated leaves were taken at 5 dpi. (**C**) Relative fungal biomass of detached maize seedling leaves. Bar indicates the standard deviation of six technical duplications and the small letters indicate a significant difference (*p* < 0.01). (**D**) Infection observation of wild type, *ΔCgGPI7*, and c*CgGPI7* at 24 h and 36 h. The red and the orange arrow indicate appressorium and invasive hyphae, respectively. Bar = 25 μm.

To determine the pathogenicity of *ΔCgGPI7*, 10-day-old maize seedlings were used for spraying or detaching inoculation. Spray inoculation showed that necrosis lesions occurred in abundance on leaves that were inoculated by the wild-type and *cCgGPI7* strains compared with those inoculated by *ΔCgGPI7* mutants (Figure 5A). Moreover, the 10 μL conidial suspension (1 × 10^5^ conidia/mL) of the corresponding strains was dropped on the epidermis of detached maize leaves and the same result showed that only slight lesions appeared on the leaves that were inoculated by *ΔCgGPI7* mutants compared with that of the wild-type and *cCgGPI7* strains (Figure 5B). qRT-PCR detection showed that there was a significant decrease in fungal biomass in the detached leaves that were infected by *ΔCgGPI7* mutants compared with the wild-type and *cCgGPI7* strains (Figure 5C). We also observed the infected process of *ΔCgGPI7* mutants, the wild-type, and *cCgGPI7* strains by inoculating the conidial suspension on maize epidermis. All the tested strains displayed the normal formation of appressoria at 24 hpi. The appressoria of wild types and *cCgGPI7* penetrated the epidermis and formed invasive hyphae in host cells, while the *ΔCgGPI7* mutants remained in the appressorial stage at 36 hpi (Figure 5D). These results indicated that *CgGPI7* mediated the appressorial penetration and was essential for the pathogenicity of *C. graminicola*.

### 2.7. CgGPI7 Affects the Cell Wall Integrity of C. graminicola

**Figure 6:**
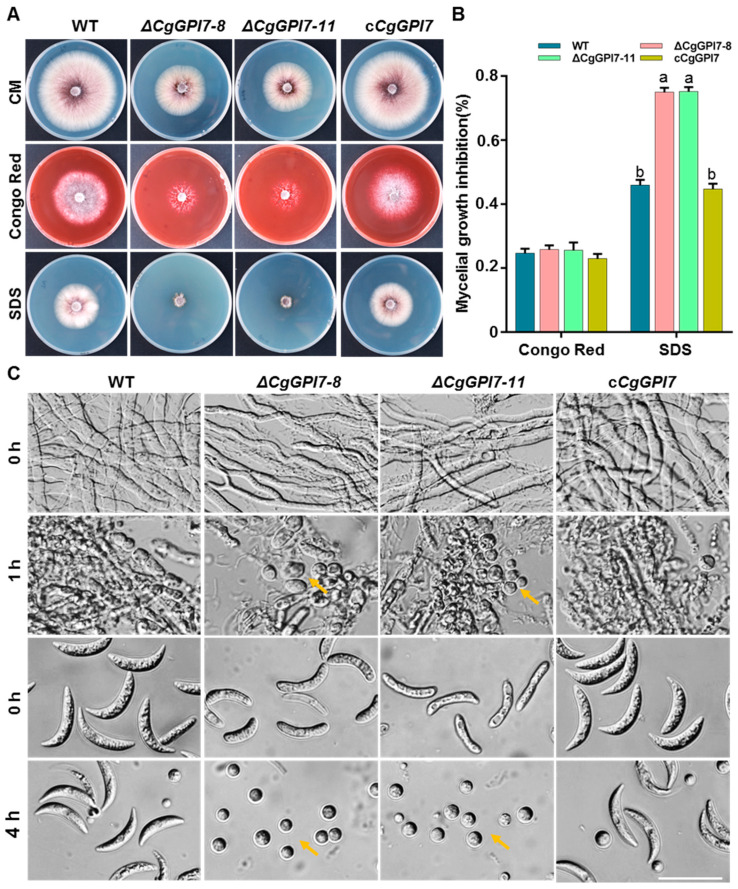
CgGPI7 is involved in cell wall integrity. (**A**) Colony morphology of wild types, *ΔCgGPI7-8*, *ΔCgGPI7-11*, and c*CgGPI7* were identified by growing these strains on a CM medium that was amended with 0.8 mg/mL (*w*/*v*) Congo Red and 0.0025% (*w*/*v*) SDS. (**B**) Statistical analysis of the inhibition rate of mycelial growth. Bar indicates the standard deviation of three replicates and the small letters indicate a significant difference (*p* < 0.01). (**C**) The mycelial and conidia of indicated strains were treated with cell wall-lysing enzymes for 1 h and 4 h, respectively. Bar = 25 μm. Orange arrows indicate the released protoplasts.

A previous study revealed that GPI7 affects cell wall integrity in *S. cerevisiae* [37]. We first determined the sensitivities of *ΔCgGPI7* to cell wall stresses, including 0.8 mg/mL Congo Red and 0.0025% sodium dodecyl sulphate (SDS), to characterize whether CgGPI7 plays a role in the cell wall integrity in *C. graminicola*. The colonies of *ΔCgGPI7* mutants cultured on CM plates containing Congo Red exhibited extraordinary sparse hyphae, although there was no significant difference in the growth inhibition rate compared with that of the wild-type and *cCgGPI7* strains. *ΔCgGPI7* mutants became more sensitive and hardly grew on CM plates containing SDS compared with that of wild-type and *cCgGPI7* strains (Figure 6A,B). We further checked the cell wall-lysing enzymes tolerance of *ΔCgGPI7* mutants, wild types, and *cCgGPI7*. More protoplasts were released from the vegetative hyphae and conidia of *ΔCgGPI7* mutants than that of the wild-type and *cCgGPI7* strains after 1 h and 4 h cell wall-lysing enzyme treatment, respectively, and confirmed that *ΔCgGPI7* mutants were more sensitive to cell wall-lysing enzymes (Figure 6C). These results suggested that *CgGPI7* may play a role in the cell wall integrity of *C. graminicola*.

### 2.8. Deletion of CgGPI7 Resulted in the Up-Regulation of Cell Wall Synthesis-Related Genes in C. graminicola

**Figure 7:**
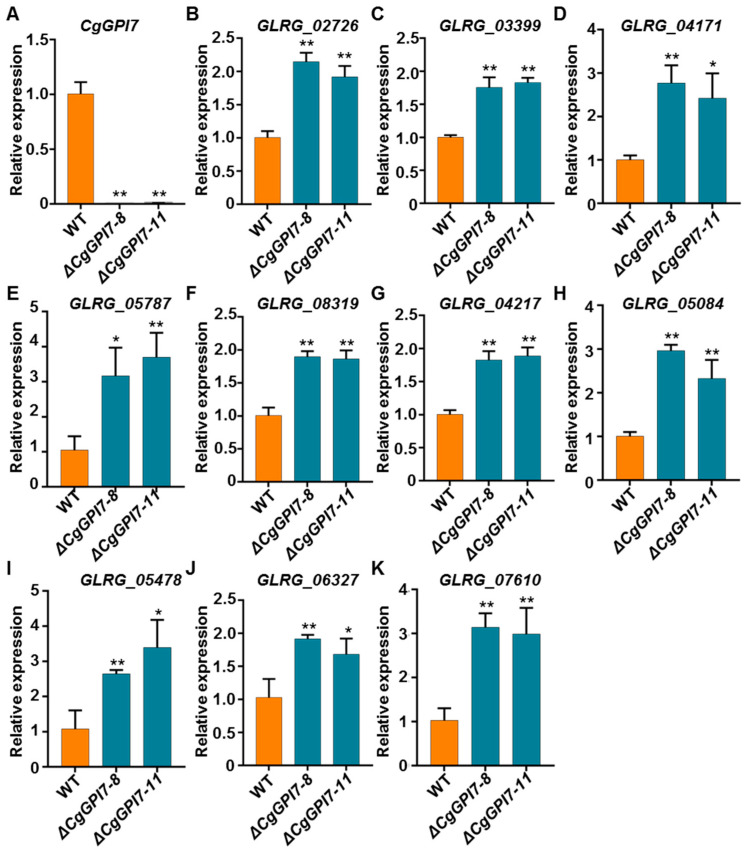
The expression of cell wall synthesis-related genes was induced in the *ΔCgGPI7*. (**A**) qRT-PCR was used to verify the expression of *CgGPI7* in wild-type, *ΔCgGPI7-8,* and *ΔCgGPI7-11* strains. (**B**–**F**) Expression of five chitin synthase genes were quantified in wild-type, *ΔCgGPI7-8,* and *ΔCgGPI7-11* strains via qRT-PCR. (**G**–**K**) Expression of five 1,3-beta-glucanosyltransferase genes were quantified in wild-type, *ΔCgGPI7-8,* and *ΔCgGPI7-11* strains via qRT-PCR. Significance analysis was analyzed using Student’s *t*-test (* *p <* 0.05, ** *p* < 0.01). The bar represents the standard deviation of the three technical duplications.

The fungal cell wall is the most important structure and is essential for cell viability, morphogenesis, and pathogenesis [38]. Previous studies reported that the inner cell wall of most fungal species consists of a core of covalently attached branched β-1,3 glucan with 3 to 4% interchain and chitin [39]. In the present study, the reduced vegetative growth rate, immature conidia morphology, defective cell wall integrity, and impaired pathogenicity happened in *ΔCgGPI7* mutants, which prompted us to speculate that *CgGPI7* might mediate the fungal cell wall synthesis. Thus, the expression levels of five chitin synthase genes (*GLRG_02726*, *GLRG_03399*, *GLRG_04171*, *GLRG_05787*, and *GLRG_08319*) and five 1,3-beta-glucanosyltransferase genes (*GLRG_04217*, *GLRG_05084*, *GLRG_06327*, *GLRG_07610*, and *GLRG_05478*) were checked in the hyphae of wild types and *ΔCgGPI7* mutants (Table S2). Firstly, we verified the expression of *CgGPI7* in wild-type, *ΔCgGPI7-8*, and *ΔCgGPI7-11* strains to confirm the disruption of *CgGPI7* (Figure 7A). Surprisingly, all the selected cell wall synthesis genes were significantly up-regulated in *ΔCgGPI7* mutants compared with the wild type (Figure 7B–K). These results suggest that *CgGPI7* may regulate the basic cell wall synthesis of *C. graminicola*, while the absence of *CgGPI7* affects the cell wall synthesis-related proteins and significantly induces the transcription of cell wall synthesis-related protein-coding genes.

## 3. Discussion

EPT family proteins were conserved in the GPI anchor biosynthesis pathway and are essential for mature GPI anchor processing. Transferring the EtN-P1 to Man1 is catalyzed by MCD4 in *S. cerevisiae* or its ortholog PIG-N in mammals (Figure S1) [23,40]. Previous studies showed that adding the specific inhibitor or the destruction of MCD4 arrested the vegetative growth and caused the accumulation of GPI anchor intermediate M2 in *S. cerevisiae* (Figure S1) [41]. Based on a space–time order, the addition of the EtN-P3 to Man3 takes precedence over the addition of EtN-P2 to Man2. Transferring the EtN-P3 to Man3 is mediated by GPI13 in *S. cerevisiae* or the ortholog PIG-O in mammals (Figure S1). Interestingly, adding EtN-P3 to Man3 is strictly dependent on GPI13 in *S. cerevisiae*, but the deletion of PIG-O does not completely abolish the addition of the GPI anchor to the proteins, which suggests that there is a minor PIG-O-independent pathway for the addition of EtN-P3 to Man3 in mammalian [42]. One speculation of the minor pathway is that the hGPI7 can transfer EtN-P3 to Man3 at the *PIGO* knockout cells [3]. In *S. cerevisiae*, the overexpression of *GPI7* or *MCD4* cannot rescue the growth of *GPI13* deletion cells [3,42]. As in the description above, the GPI7/hGPI7 is responsible for transferring EtN-P2 to Man2. The deletion of GPI7 caused the defect on EtN-P2 transferring, impaired the cell wall biosynthesis, and slowed down the transport rate of GPI proteins from ER to Golgi compared with the wild type [3,43]. All the above results demonstrated the important biochemical roles of the three EPT family proteins in GPI anchor biosynthesis and aroused our interest in exploring the corresponding roles in *C. graminicola*. 

In this study, we identified three putative EPT family genes (*CgMCD4*, *CgGPI7*, and *CgGPI13*) in *C. graminicola*. Homologous searching showed that the proteins encoded by these genes were conserved in other filamentous pathogenic fungi and proved that GPI anchoring also was a common post-translational modification in these fungi. The multi-alignment of the amino acid sequences of CgMCD4, CgGPI7, and CgGPI13 exhibited only a total identity of 21.06%, which suggested a functional differentiation of these three proteins in *C. graminicola*. Correspondingly, in our study, except for the *CgGPI7*, we could not obtain the knockout mutants of *CgMCD4* and *CgGPI13*, which is consistent with the results of the previous studies of *S. cerevisiae* and demonstrated that *CgMCD4* and *CgGPI13* mediated GPI anchor biosynthesis or that unknown functions in *C. graminicola* might be indispensable to fungal survival [22,23]. Comparatively, *CgGPI7* might be weaker for *C. graminicola* survival, even though *ΔCgGPI7* mutants exhibited serious defects in vegetative growth, conidia morphology development, conidiation, pathogenicity, and cell wall integrity. In the previous study, RNA interference results showed that GPI anchor synthesis-related genes *CgGPI12*, *CgGAA1,* and *CgGPI8* (Table S2) are indispensable for vegetative development and the pathogenicity of *C. graminicola* [36]. Our results were consistent with the previous results and confirmed that GPI anchoring was essential for development and pathogenicity.

The fungal cell wall is required for cell viability, morphogenesis, surrounding environment response, and pathogenicity. Except for the basic polysaccharide scaffolding, the cell wall proteins mediated the cell wall biosynthesis and remodeling [39]. Cell wall proteins are frequently modified by GPI anchoring. Liu et.al used the FLAER-specific staining method and proved that the deletion of *GPI7* caused the significant reduction of GPI-anchored proteins on the plasma or cell wall region of conidia, hyphae, appressorium, and invasive hyphae in *M. oryzae*, and found that the *ΔCgGPI7* mutants were easily recognized by the host immune system because the defect GPI anchor proteins caused the exposure of cell wall chitin and β-1,3-glucans during the infection process, illuminating the GPI anchoring-mediated pathogen–host interaction using the modal *M. oryze*-Rice system [12]. In our study, we also found that the deletion of *CgGPI7* caused the cell wall integrity defect of *C. graminicola*. The abnormal conidia morphology development and more cell wall-lysing enzyme sensitivity demonstrated that *CgGPI7* deletion influenced the GPI-anchored-protein-mediated cell wall biosynthesis and remodeling in *C. graminicola*.

As the essential structure for cell survival, the fungal cell wall employs multiple ways to participate in the cell wall salvage response when the environment changes or is exposed to cell wall destructive agents. Therefore, the integrity chitin-β-1,3-glucan cell wall scaffold must be monitored and regulated constantly to enable the maintenance or restoration of dynamically changing cell walls [39]. Among four cell wall integrity maintenance pathways, the best characterized one is the protein kinase C pathway regulated by the highly glycosylated integral membrane sensors Mid2 and Mtl1 in *S. cerevisiae*. The perturbations were perceived by the sensors in the cell wall and transduced the MAPK cascade signals to activate transcription factors to regulate the expression cell wall biosynthesis-related genes [44]. The other cell wall biosynthesis pathways include the Ca^2+^/calcineurin pathway, the HOG pathway, and the pH-sensing RIM101 pathway. These pathways mediated the activation of cell wall compensatory or salvage, resulting in elevated chitin levels and an increase in the number of GPI proteins [45,46]. In our study, we found that the deletion of *CgGPI7* significantly induced the up-regulated expression of chitin synthases and β-1,3-glucanosyltransferases in *C. graminicola***,** which indirectly proved that the disturbance of endogenic GPI anchor synthesis could affect the cell wall integrity and activate the cell wall salvage response. 

## 4. Materials and Methods

### 4.1. Fungal Strains and Plant Culture Conditions

The *C. graminicola* strain CgM2 was used as the wild type to generate the specific transformant strains. All the *C. graminicola* strains in this study were grown on complete medium (CM: YEAST EXTRACT, LP0021, OXOID; Casamino Acids, 8197978, Bacto^TM^; N-Z-Amine, C0626-500G, SIGMA; Agar, CA1331-1KG, Coolaber) agar plates, potato dextrose agar (PDA, HB0233-5, hopebio) plates or oatmeal agar (OA: oatmeal, SEAMILD; Agar, CA1331-1KG, Coolaber) plates at 25 °C. The maize seedling plants used for the fungal pathogenicity test were grown in the growth chamber (25 °C, 14/10 days/night, 85% humidity).

### 4.2. Bioinformatic and Phylogenetic Analysis 

The GPI anchor synthesis pathway-related proteins in *C.* *graminicola* were searched in BLAST against in the Ensemble Fungi database (http://fungi.ensembl.org/Colletotrichum_graminicola/Info/Index, accessed on 1 December 2020) using *S. cerevisiae*-corresponding homologs as queries. The homologous proteins of three GPI ethanolamine phosphate transferases, GPI7, GPI13, and MCD4, from other fungal species were also found in the Ensemble Fungi database (http://fungi.ensembl.org/index.html, accessed on 1 December 2020). The phylogenetic tree was produced using MEGA7.0 software with the neighbor-joining method and the multiple amino acid sequence alignment was constructed using DNAMAN software. The functional domains of GPI7, GPI13, and MCD4 homologs were predicted using the SMART online tool (http://smart.embl-heidelberg.de/, accessed on 1 December 2020).

### 4.3. Gene Disruption and Complementation

Gene disruption was performed through a split PCR strategy, as previously described [12] (Figure S5A). Additionally, the hygromycin B (400052-20mL, MERCK) phosphotransferase gene was used as the target gene replaced element for deletion mutant selection. The whole *CgGPI7* gene, including a 1.5 kb native promoter region and a 0.5 kb terminator region, was amplified and cloned into pGTN vector to make the complement construct (Figure S5A). The resulting construct was transformed into the protoplast of the *ΔCgGPI7* mutant, and the G418 (G6021-5g, MACKLIN) resistance was used for transformants selection. Primers were used to identify the gene disruption and complementation transformants listed in Table S1 and the corresponding results are shown in Figure S5. 

### 4.4. Biological Phenotypic Analysis

To analyze the fungal growth rate, the mycelial blocks of the wild type, *ΔCgGPI7-8*, *ΔCgGPI7-11*, and *cCgGPI7* were cultured on CM and PDA plates with three independent replicates, and the colony diameters were measured at 120 h post-inoculation (hpi). For the conidiation test, the mycelial blocks of indicated strains were inoculated on OA plates with three independent replicates, and the conidiation was measured after 14 days. Each plate was washed with 10 mL ddH_2_O three times to create a full elution of the conidia. The resulting conidia suspensions were counted under a microscope with a hemocytometer and counted three times to average the final value. The morphology of conidia was observed under a Leica DM6B fluorescence microscope and the conidia length and width were measured using Image J software.

### 4.5. Cell Wall Integrity Analysis

To test the cell wall stress response, the mycelial blocks of the wild type, *ΔCgGPI7-8*, *ΔCgGPI7-11*, and *cCgGPI7* were cultured on CM plates that contained 0.8 mg/mL (*w*/*v*) Congo Red or 0.0025% (*w*/*v*) sodium dodecyl sulphate (SDS). The equal amounts of mycelia from different strains were treated with lysing enzymes (L1412, Sigma) for 1 h, and the same concentrations of conidia were treated with lysing enzymes for 4 h to test the cell wall integrity of the specific strains.

### 4.6. Subcellular Localization Analysis of CgGPI7

The subcellular localization of CgGPI7 was predicted using PSORT software (https://www.genscript.com/psort.html, accessed on 1 February 2021). To verify the ER localization of CgGPI7, the ER maker *RFP-HDEL* was co-transformed with *CgGPI7-GFP* into the protoplast of the *ΔCgGPI7* mutant. The transformants were observed under a Leica DM6B fluorescence microscope for subcellular localization analysis.

### 4.7. Pathogenicity Analysis

To identify the pathogenicity of different fungal strains, 10 days old maize seedlings were sprayed with conidial suspensions (1 × 10^5^ conidia/mL). Moreover, we also used the detached inoculation method that involved dropping conidia droplets on detached maize seedling leaves to identify the pathogenicity of the different fungal strains. The inoculated plants or leaves were incubated at 25 °C with full humidity and the disease lesions were measured at 5 dpi. The initial infection process was observed by inoculating the conidia droplets on the lower maize leaves and the inoculated epidermis was torn down to be observed at 24 hpi and 36 hpi.

### 4.8. qRT-PCR Assays

To analyze the expression of *CgGPI7* at different development stages, the samples including conidia, vegetative hyphae, and the maize seedling leaves after being infected by *C. graminicola* were collected. Conidia were collected from 14-day OA plates. Vegetative hyphae were cultured in 100 mL liquid CM medium at 28 °C (180 rpm) for 36 h and then were harvested. For infected maize seedling leaf collection, the 10-day-old maize seedlings were sprayed with conidial suspensions (1 × 10^5^ conidia/mL) and the three independent second leaves at 12 h, 24 h, 36 h, 48 h, 60 h, 72 h, and 96 h post-inoculation were collected.

To analyze the cell wall synthesis-related gene expression in wild-type and *ΔCgGPI7* strains, the vegetative hyphae of CgM2, *ΔCgGPI7-8*, and *ΔCgGPI7-11* were cultured in 100 mL liquid CM medium at 28 °C (180 rpm) for 36 h and then were harvested.

The above samples were ground into powder in liquid nitrogen, and then total RNA was extracted with TRIzol reagent (318307, Ambion) and the cDNA was synthesized using a commercial kit (R312-01, Vazyme). The gene transcriptional levels were detected using an ABI7500 FAST Real-Time PCR Detection System. The fungal *Histone3* (*H3*) and maize *Actin* internal reference genes were used for the data normalization. The 2^−ΔΔCT^ method was used to calculate the relative expression levels with three technical repeats. Gene-specific primers for qRT-PCR (Quantitative RT-PCR) are listed in Table S1.

### 4.9. Statistical Analysis Method

Data analysis was performed using SPSS25 software. A one-way ANOVA, Fisher’s LSD test, and Student’s *t*-test were used for statistical analysis (* *p* < 0.05, ** *p* < 0.01). All the charts in the article were made using GraphPad Prism 7.

## 5. Conclusions

In summary, we identified the conservation of GPI anchor biosynthesis pathways. A functional study was focus on the three putative EPT family genes (*CgMCD4*, *CgGPI13*, and *CgGPI7*) in *C. graminicola*. The results demonstrated that *CgMCD4* and *CgGPI13* might be indispensable for the survival of *C. graminicola*. Additionally, the *CgGPI7* is essential for the normal vegetative growth, conidia development, cell wall integrity, and pathogenicity of *C. gramincola*. More importantly, we found that CgGPI7-mediated GPI anchor modification defection might be a new element that induces the cell wall salvage response to control the vegetative growth and pathogenicity in *C. gramincola*.

## Data Availability

The data presented in this study are available on request from the corresponding authors.

## Supplementary Materials

The following supporting information can be downloaded at: https://www.mdpi.com/article/10.3390/ijms23062985/s1.
