# Supplementary material for: Glycosylphosphatidylinositol Anchor Biosynthesis Pathway-Related Protein GPI7 Is Required for the Vegetative Growth and Pathogenicity of Colletotrichum graminicola
Source: Int J Mol Sci. 2022 Mar 10;23(6):2985. doi: 10.3390/ijms23062985 (PMC8949851; doi:10.3390/ijms23062985)
Supplement: Supplementary file 1 [file ijms-23-02985-s001.zip › Supplemental Figure S1-S5.pdf]

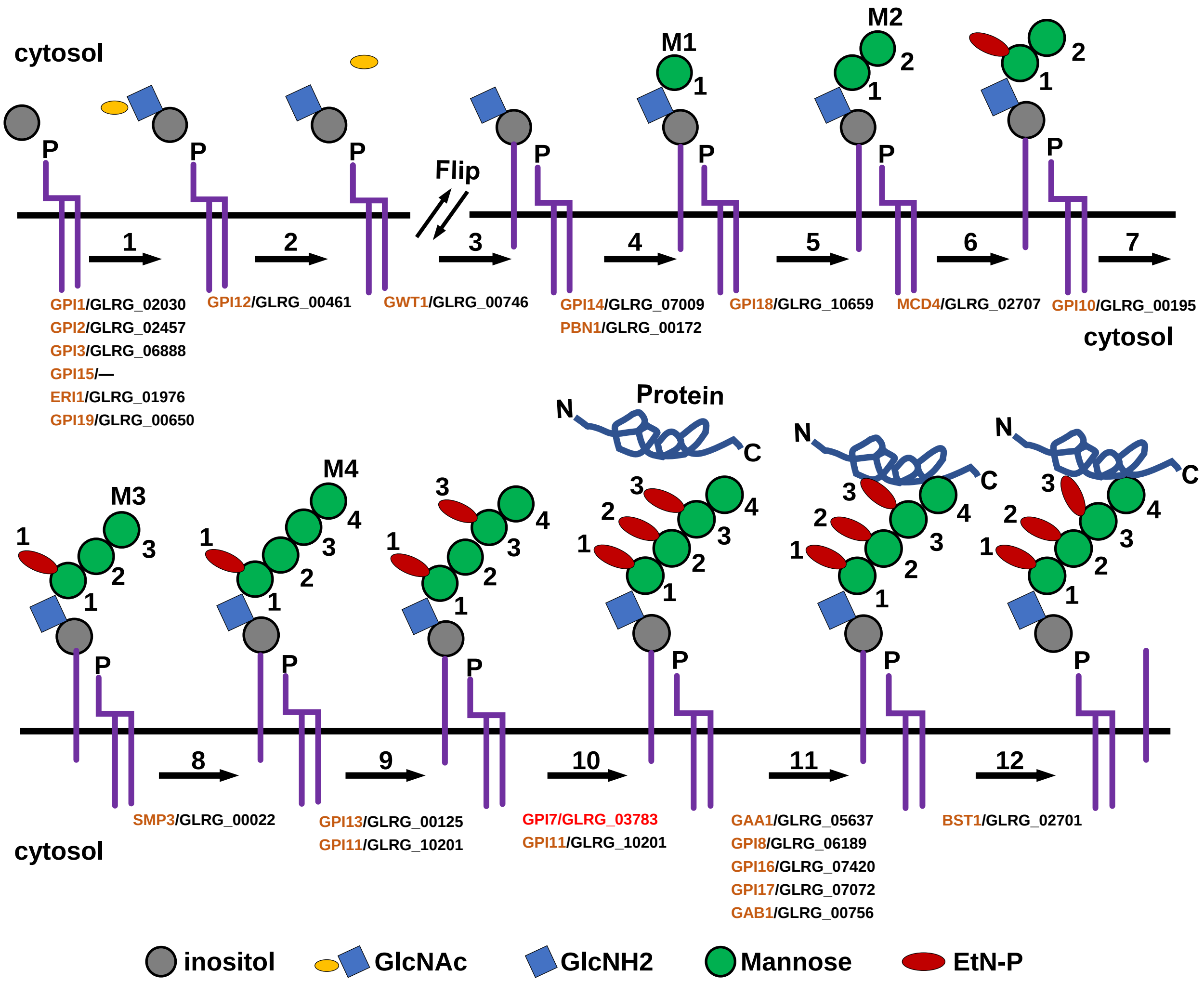

Supplemental Figure S1: Putative GPI-anchor synthesis pathway in *C.graminicolla*. The figure was drawn by referring to *S.cerevisiae*. The GPI-anchor synthesis pathway proteins in *S.cerevisiae* and the corresponding homologous proteins found in *C.graminicolla* are shown. Number 1 to 12 indicated the biosynthetic steps. $GlcNH_2$, Glucosamine; GlcNAc, N-Acetyl-Glucosamine; Man, Mannose; EtN-P, phosphorylethanolamine; inositol-P, phosphatidylinositol.

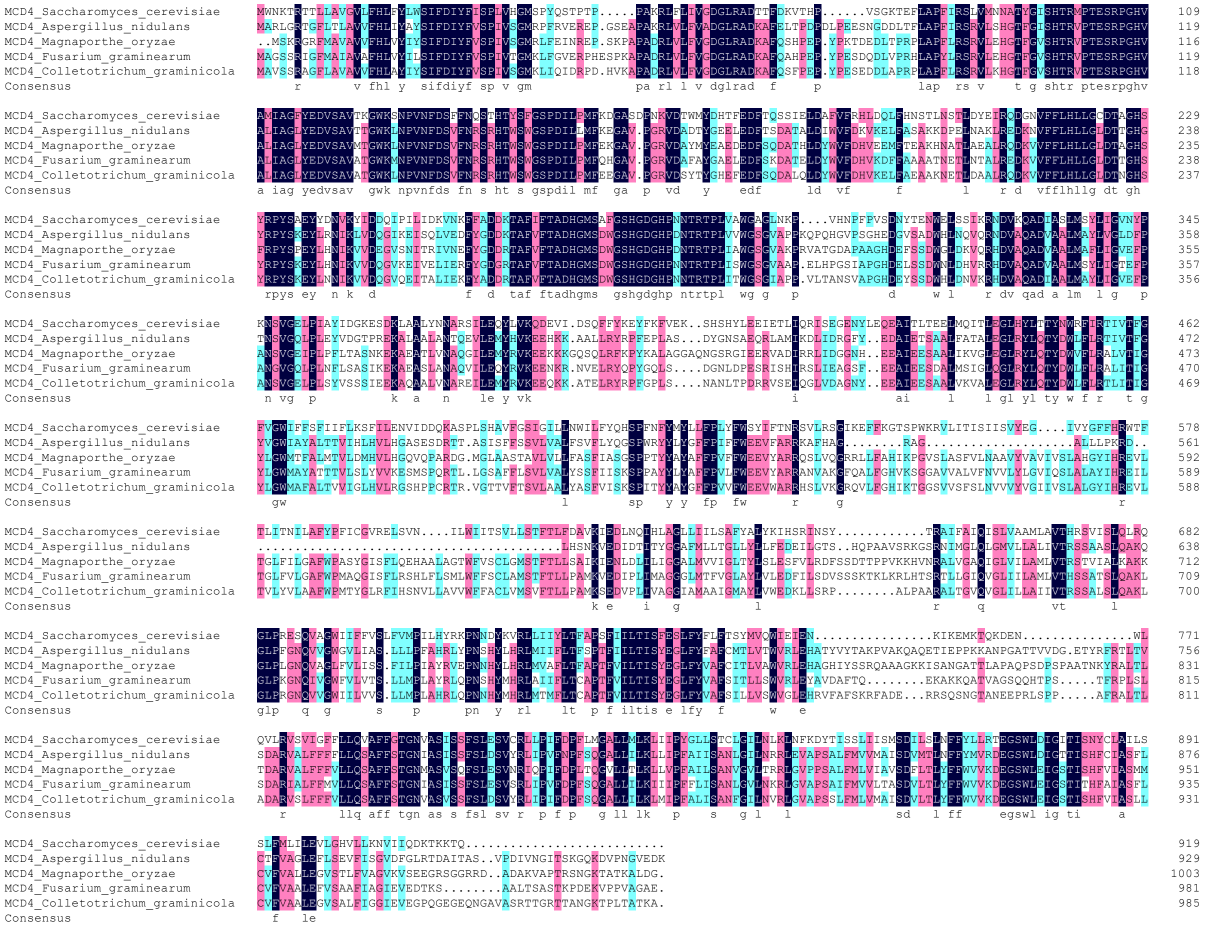

Supplemental Figure S2: Multiple alignment of MCD4 from different fungi species. Homologous proteins of MCD4 from 5 different fungi species and performed multiple alignment. Total sequence identity is 67.09%.

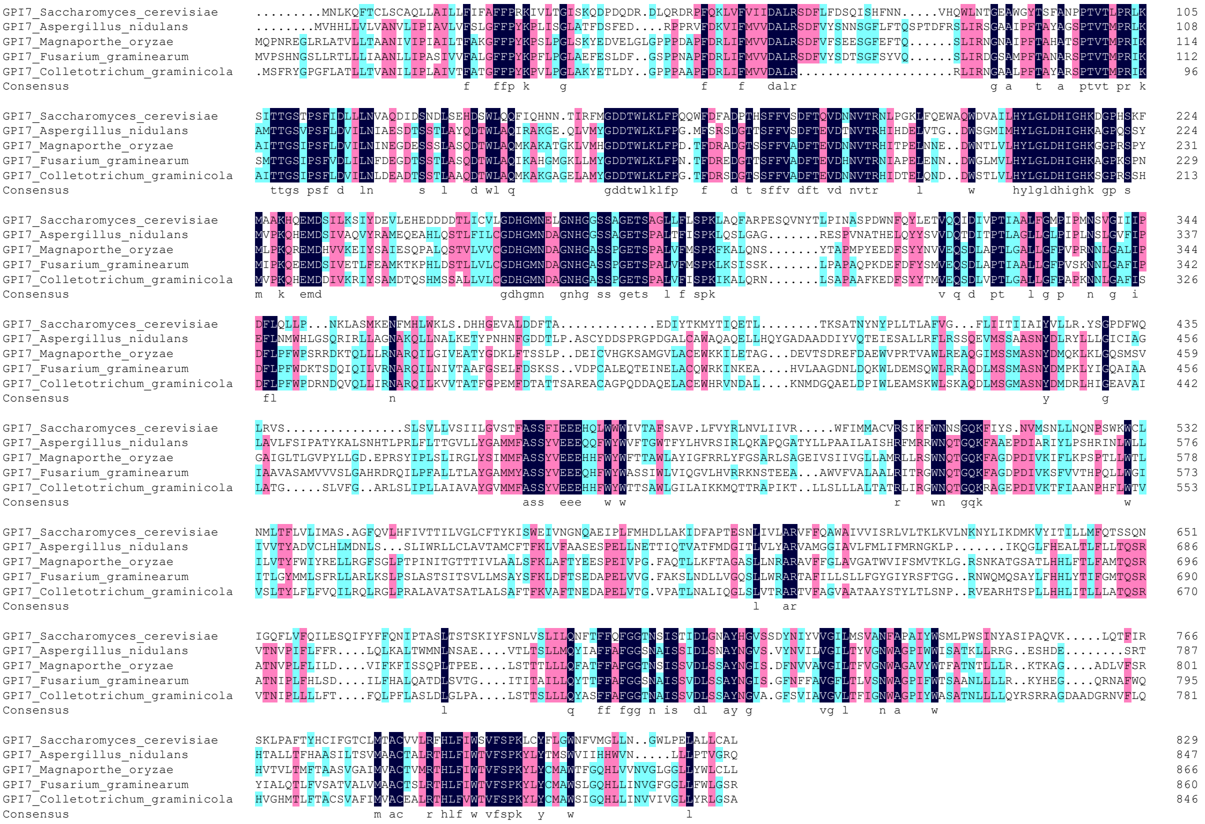

Supplemental Figure S3: Multiple alignment of GPI7 from different fungi species. Homologous proteins of GPI7 from 5 different fungi species and performed multiple alignment. Total sequence identity is 57.15%.

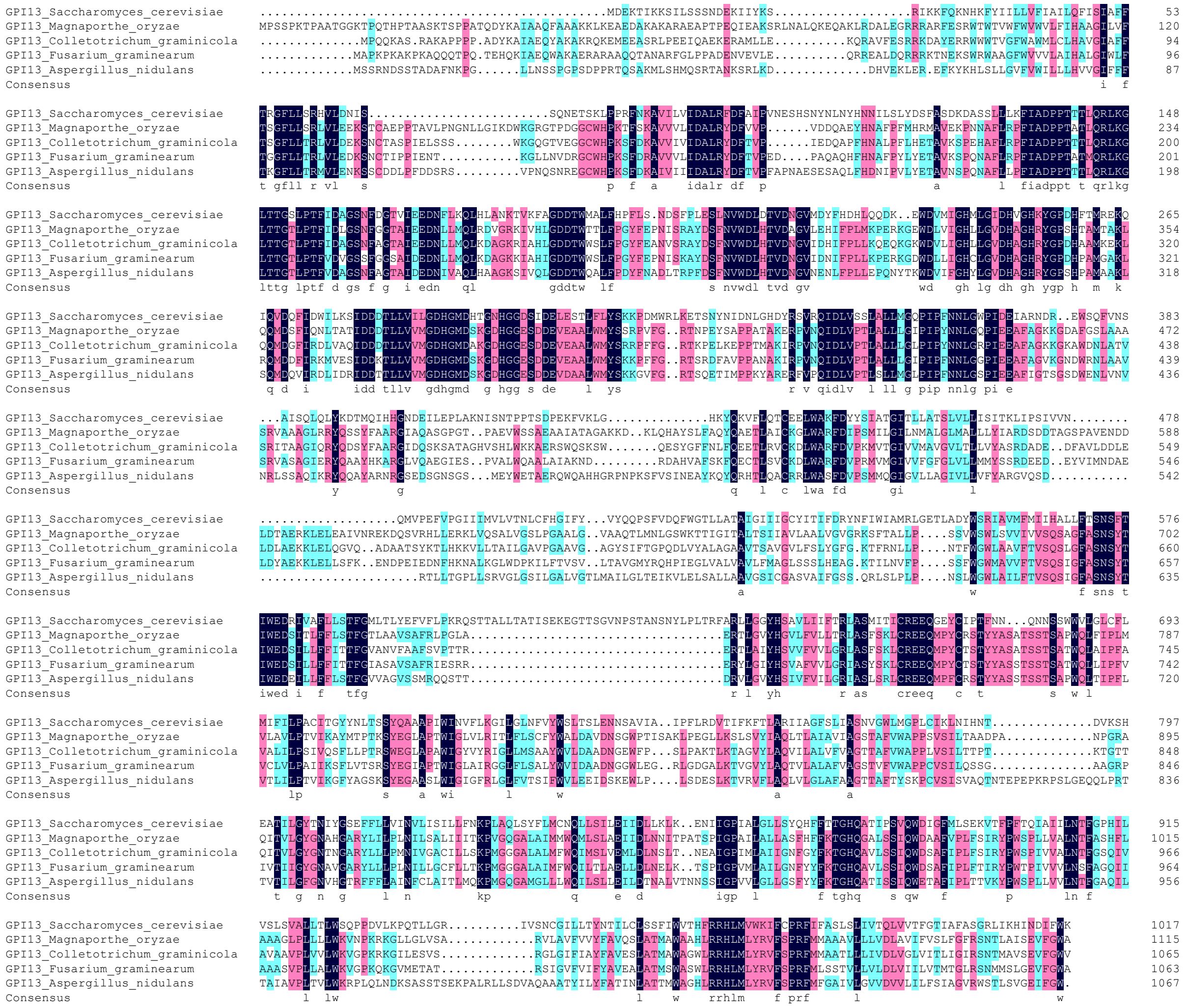

Supplemental Figure S4: Multiple alignment of GPI13 from different fungi species. Homologous proteins of GPI13 from 5 different fungi species and performed multiple alignment. Total sequence identity is 55.08%.

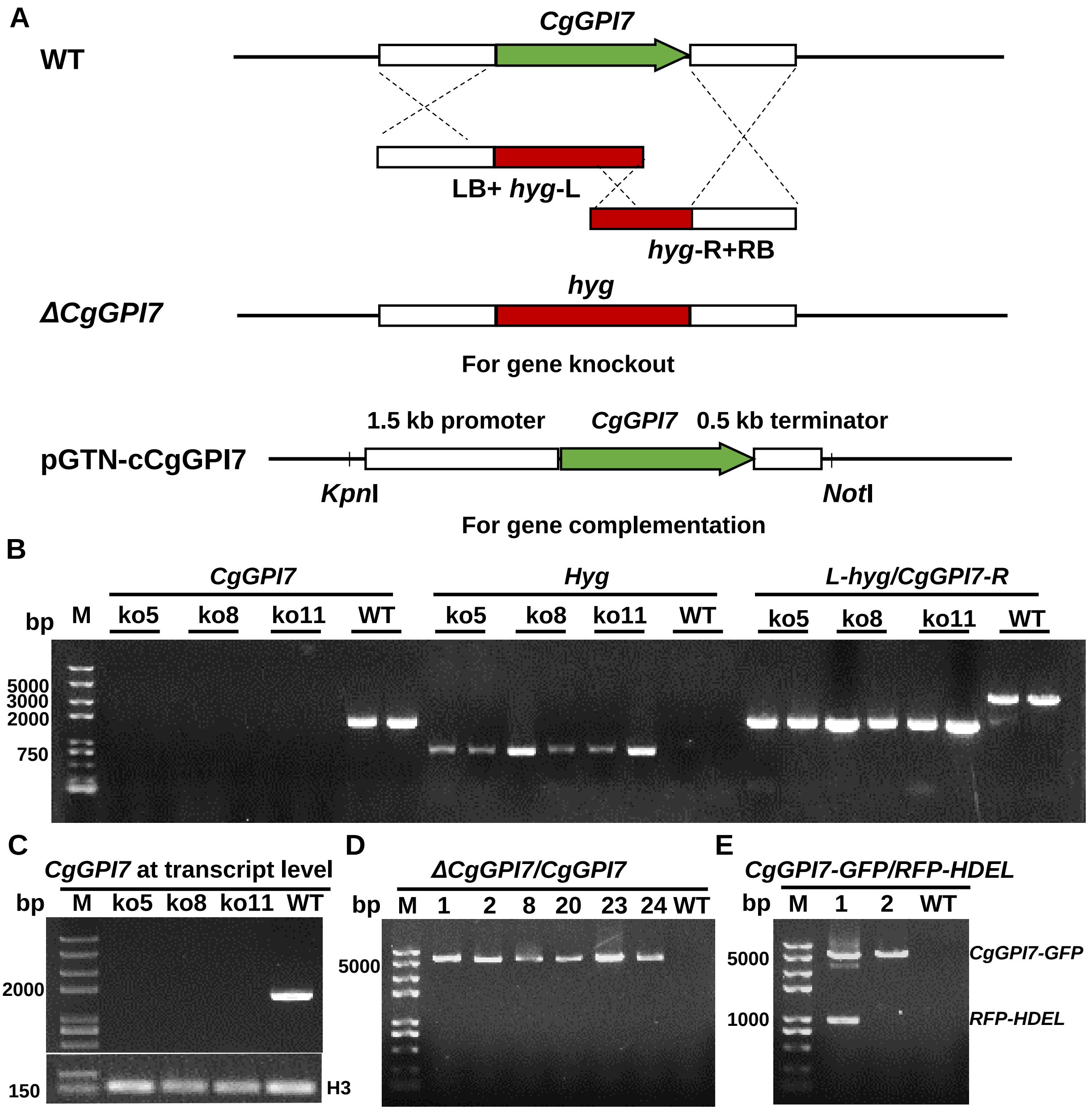

Supplemental Figure S5: Gene knockout and complementation of *CgGPI7* in *C.graminicolla*. (A) The *CgGPI7* gene knockout and complementation schematics. Gene knockout through a split-marker approach. And gene complementation by native promoter. (B) Verification of three *CgGPI7* knockout mutants by PCR. *CgGPI7* gene fragments couldn't be amplificated in *ΔCgGPI7* mutants. Hygromycin resistance gene fragments could be amplificated in *ΔCgGPI7* mutants. In situ amplification shown the smaller bands were amplificated in *ΔCgGPI7* mutants which contain the replaced *hyg* fragments.(C) Verification of *CgGPI7* knockout mutants at the transcriptional level. (D) Verification of *CgGPI7* gene complementation strains by PCR. The fusion fragments contain native promoter, ORF and terminator of *CgGPI7* were amplificated in complementation strains using the vector primer. (E) Verification of CgGPI7-GFP and RFP-HDEL co-transformant strains by PCR. There are two bands including *RFP-HDEL* and *CgGPI7-GFP* can be amplified in correct strain by same vector primer.
