# Supplementary material for: Glycosylphosphatidylinositol Anchor Biosynthesis Pathway-Related Protein GPI7 Is Required for the Vegetative Growth and Pathogenicity of Colletotrichum graminicola
Source: Int J Mol Sci. 2022 Mar 10;23(6):2985. doi: 10.3390/ijms23062985 (PMC8949851; doi:10.3390/ijms23062985)
Supplement: Supplementary file 1 [file ijms-23-02985-s001.zip › Supplemental Table S1-S2.pdf]

Supplemental Table S1. Primers used in this study

Primer name	Sequence (5'-3')
Knockout mutant strains construction primers	
Hyg-F	CTTGGCTGGAGCTAGTGGAGGT
Hyg-R	CCCGGTCGGCATCTACTCTATTC
HY-F	CGTTGCAAGACCTGCCTGAA
YG-R	GGATGCCTCCGCTCGAAGTA
MCD4-LF1	GCGAAAGTGGCAGCATTGACTA
MCD4-LR1	ACCTCCACTAGCTCCAGCCAAGAATGGTACTGTCCTCGGCTCTCG
MCD4-LF2	GTATCTTGGTGGCTCGGCTCA
MCD4-RF1	GAATAGAGTAGATGCCGACCGGGGCTCGTGGTTAGAGATTGGCTCC
MCD4-RR1	TGAATGGGTTGGTCAAGGTGC
MCD4-RR2	GGTGGGTGCCGAGAATGATAA
GPI13-LF1	CCATTGTTGGAGAGGGCTACGAT
GPI13-LR1	ACCTCCACTAGCTCCAGCCAAGGGGTGATGTGGGCAGGAAGTAA
GPI13-LF2	ATAGCCGTCGCTTTCCAGTGG
GPI13-RF1	GAATAGAGTAGATGCCGACCGGGCGATGGCTGTCAGTGAGGTCTTT
GPI13-RR1	CCTACGAAGAACAACGGCATACG
GPI13-RR2	GGATTGAAGGAGGTGATGAAGGC
GPI7-LF1	AAATGGGAGGAGACCACACAGC
GPI7-LR1	ACCTCCACTAGCTCCAGCCAAGGGGTTTCAACGACTGGAAGAGG
GPI7-LF2	TCTCGCACAGTCACTTTCACCC
GPI7-RF1	GAATAGAGTAGATGCCGACCGGG
GPI7-RR1	GATTGAAGGCGAAGAGGACGAA
GPI7-RR2	CGAGCAAAGGCTGTCAAGGAG
**Knockout mutant strain checking primers**	
MCD4-F	CCAGTCCTTTCCAGAACCCTACC
MCD4-R	ACAAGTGAATGACGCCGAGC
GPI13-F	TCATTGACGCTCTGCGATACG
GPI13-R	CGAGTGACTGGCTAACCGTGAA
GPI7-F	GAATCTTGATGAAGCCGACACG
GPI7-R	TGAGCCACTGCGGGATGTTA
GPI7-L-F	TCATTCCCCCTTCAACGTGC
GPI7-R-R	GGATCATGGGACAAGGGAGC
hyg-f	CAGCTTCGATGTAGGAGGGC
hyg-r	CGAAATTGCCGTCAACCAAG
**Complementary and subcellular localization strains construction primers**	

C-GPI7-F	AGGGAACAAAAGCTGGGTACCTCTCGCACAGTCACTTTCACCC
C-GPI7-R	AGTAACGTTAAGTGCGGCCGCCAGGCTGATCTCCGACACAACT
Sub-GPI7-R	CATTCTAGAACTAGTGGATCCCGCAGATCCCAGTCGGTACAAGA
**Complementary and subcellular localization strains checking primers**	
pGTN-cx-F	CATTAGGCACCCCAGGCTTT
pGTN-cx-R	TCGTGACCA CCCTGACCTAC
**qRT-PCR primers**	
GPI7-qPCR-F	TCTGCGGAGACCACGGTATG
GPI7-qPCR-R	GTCCTCTTTGAACGCAGCAGG
GLRG_02726-F	CGTTGGTTCTTGGGTTTCCTC
GLRG_02726-R	CGTGGTGATGATGGCGAGTA
GLRG_03399-F	GCGGCAGCGAGGAGTTTAT
GLRG_03399-R	CAGCAGCACCTTGTCTTCGTT
GLRG_04171-F	GGTTCTTCCAGGCATTCGG
GLRG_04171-R	TGGGTTGAGGAGGTGCTTTC
GLRG_05787-F	TGGTTGGCAGAAGATTGTGGT
GLRG_05787-R	TGAGGTCAGAGTCAAGGGACAC
GLRG_08319-F	GCGTTCAGCCGACTTCAGT
GLRG_08319-R	AATCTCCATCCAGCACCGTT
GLRG_04217-F	AGCCGAAACCACCTTGACC
GLRG_04217-R	CATCGTCGCTACCGCAGTT
GLRG_05084-F	GCCGACATTGCTGAACTGC
GLRG_05084-R	GGTGAAGAAGATAGGCACGGG
GLRG_05478-F	AGGCGGAAGCGATGAAGA
GLRG_05478-R	TCCCTGAACCTCCCAGTTAGAG
GLRG_06327-F	CCCATTCCCGTCGGTTACT
GLRG_06327-R	TTGAAGTCAGAGTCGCCGC
GLRG_07610-F	TGCGTGCTTGTCCAATCC
GLRG_07610-R	TGTGCTTGCGGTCGTGTT
CgH3-F	TCAAGAAGCCTCACCGCTACAA
CgH3-F	GAAGTCCTGGGCAATCTCACG
ZmActin-F	GATTCCTGGGATTGCCGAT
ZmActin-R	TCTGCTGCTGAAAAGTGCTGAG

Supplemental Table S2. The genes involved in this study

Colletotrichum graminicola	*Saccharomyces cerevisiae*	Annotation of putative protein
GLRG_02030	*GPI1*	Phosphatidylinositol N-acetylglucosaminyltransferase subunit
GLRG_02457	*GPI2*	Phosphatidylinositol N-acetylglucosaminyltransferase subunit
GLRG_06888	*GPI3*	Phosphatidylinositol N-acetylglucosaminyltransferase subunit
—	*GPI15*	Phosphatidylinositol N-acetylglucosaminyltransferase subunit
GLRG_01976	*ERI1*	Phosphatidylinositol N-acetylglucosaminyltransferase subunit
GLRG_00650	*GPI19*	Phosphatidylinositol N-acetylglucosaminyltransferase subunit
GLRG_00461	*GPI12*	N-acetylglucosaminyl-phosphatidylinositol de-N-acetylase
GLRG_00746	*GWT1*	GPI-anchored wall transfer protein
GLRG_07009	*GPI14*	GPI mannosyltransferase
GLRG_00172	*PBN1*	Protein PBN1 that probably acts by stabilizing the mannosyltransferase
GLRG_10659	*GPI18*	GPI mannosyltransferase 2
GLRG_02707	*MCD4*	GPI ethanolamine phosphate transferase 1
GLRG_00195	*GPI10*	GPI mannosyltransferase 3
GLRG_00022	*SMP3*	GPI mannosyltransferase 4
GLRG_00125	*GPI13*	GPI ethanolamine phosphate transferase 3
GLRG_10201	*GPI11*	Glycosylphosphatidylinositol anchor biosynthesis protein 11
GLRG_03783	*GPI7/LAS21*	ethanolamine phosphate transferase 2
GLRG_05637	*GAA1*	GPI transamidase component
GLRG_06189	*GPI8*	GPI-anchor transamidase
GLRG_07420	*GPI16*	GPI transamidase component
GLRG_07072	*GPI17*	GPI transamidase component
GLRG_00756	*GAB1*	GPI transamidase component
GLRG_02701	*BST1*	GPI inositol-deacylase
GLRG_04171	*CHS1*	Chitin synthase I
GLRG_03399	*CHS2*	Chitin synthase II
GLRG_05787	*CHS2*	Chitin synthase II
GLRG_02726	*CHS3*	Chitin synthase III
GLRG_08319	*CHS3*	Chitin synthase III
GLRG_05478	*GAS1*	1,3-beta-glucanosyltransferase

GLRG_05084	*GAS2*	1,3-beta-glucanosyltransferase
GLRG_06327	*GAS3*	1,3-beta-glucanosyltransferase
GLRG_07610	*GAS4*	1,3-beta-glucanosyltransferase
GLRG_04217	*GAS5*	1,3-beta-glucanosyltransferase
